# Reclaimed water as a reservoir of antibiotic resistance genes: distribution system and irrigation implications

**DOI:** 10.3389/fmicb.2013.00130

**Published:** 2013-05-28

**Authors:** Nicole Fahrenfeld, Yanjun Ma, Maureen O’Brien, Amy Pruden

**Affiliations:** ^1^Department of Civil and Environmental Engineering, Virginia TechBlacksburg, VA, USA; ^2^Department of Civil and Environmental Engineering, Colorado School of MinesGolden, CO, USA

**Keywords:** antibiotic resistance genes, water reuse, reclaimed water distribution systems, irrigation

## Abstract

Treated wastewater is increasingly being reused to achieve sustainable water management in arid regions. The objective of this study was to quantify the distribution of antibiotic resistance genes (ARGs) in recycled water, particularly after it has passed through the distribution system, and to consider point-of-use implications for soil irrigation. Three separate reclaimed wastewater distribution systems in the western U.S. were examined. Quantitative polymerase chain reaction (qPCR) was used to quantify ARGs corresponding to resistance to sulfonamides (*sul*1, *sul*2), macrolides (*erm*F), tetracycline [*tet*(A), *tet*(O)], glycopeptides (*van*A), and methicillin (*mec*A), in addition to genes present in waterborne pathogens *Legionella pneumophila* (L*mip*), *Escherichia coli* (*gad*AB), and *Pseudomonas aeruginosa* (*ecf*x, *gyr*B). In a parallel lab study, the effect of irrigating an agricultural soil with secondary, chlorinated, or dechlorinated wastewater effluent was examined in batch microcosms. A broader range of ARGs were detected after the reclaimed water passed through the distribution systems, highlighting the importance of considering bacterial re-growth and the overall water quality at the point of use (POU). Screening for pathogens with qPCR indicated presence of L*mip* and *gad*AB genes, but not *ecf*x or *gyr*B. In the lab study, chlorination was observed to reduce 16S rRNA and *sul*2 gene copies in the wastewater effluent, while dechlorination had no apparent effect. ARGs levels did not change with time in soil slurries incubated after a single irrigation event with any of the effluents. However, when irrigated repeatedly with secondary wastewater effluent (not chlorinated or dechlorinated), elevated levels of *sul*1 and *sul*2 were observed. This study suggests that reclaimed water may be an important reservoir of ARGs, especially at the POU, and that attention should be directed toward the fate of ARGs in irrigation water and the implications for human health.

## INTRODUCTION

Water reuse is an increasingly common sustainable water management practice motivated by climate change, urbanization, energy efficiency, and environmental protection (US Environmental Protection Agency [USEPA], 2012). Reclaimed or recycled wastewater is treated by municipalities for a variety of purposes, including non-potable urban reuse (Grant et al., 2012; USEPA, 2012). In the United States Environmental Protection Agency (USEPA) guidelines on water reuse, the presence of antibiotics as trace organic contaminants in wastewater is noted and a need for more information is acknowledged to reduce the proliferation of antibiotic resistance and protect public health (USEPA, 2012).

Antibiotic resistance proliferation is currently outpacing the development of novel antibiotics, calling for effective strategies to mitigate the spread of antibiotic resistance (Carlet et al., 2012). Bacterial resistance to antibiotics is partially conferred through antibiotic resistance genes (ARGs), which code for specific antimicrobial functions such as efflux pumps (Webber and Piddock, 2003). ARG contamination has been quantified in a variety of environmentally relevant matrices, including wastewater treatment plant (WWTP) effluent, which is known to contribute to ARG loadings in surface waters (Pruden et al., 2013; Storteboom et al., 2010; LaPara et al., 2011). Some states require chlorine or UV disinfection for reused water (USEPA, 2012) and certain disinfectants (free chlorine, O_3_, and UV) are capable of reacting with nucleic acids during treatment and therefore may potentially reduce ARGs, as recently reviewed by Dodd (2012). However, McKinney and Pruden (2012) recently demonstrated in a controlled lab study that typical UV doses applied at WWTPs are capable of reducing antibiotic resistant strains of bacteria, but not ARGs. Others have noted little reduction in ARGs following UV effluent treatment in full-scale WWTPs (Auerbach et al., 2007; Kim et al., 2010).

While WWTPs are now well-established as a reservoir of ARGs (Auerbach et al., 2007; Kim et al., 2010; Czekalski et al., 2012), and some have considered effect of irrigation with reclaimed water (McLain and Williams, 2012; Negreanu et al., 2012), there is a void of studies focused on the potential for re-growth in treated wastewater distribution systems (“purple” pipes). In one study examining soil irrigated with treated wastewater, no differences in the microbiome or ARG levels were observed compared to soil irrigated with fresh water (Negreanu et al., 2012). In fact, in Llobregat (NE Spain), reclaimed water emitted to a river had lower concentrations of indicator organisms than the stream water (Rubiano et al., 2012). In contrast, re-growth of indicator organisms has been observed between the point of entry (POE) and point of use (POU) in reclaimed water systems (Ryu et al., 2005), raising the question of whether ARGs can also increase during distribution. In a study examining drinking water distribution systems, antibiotic resistant bacteria were shown to decrease between POE and POU, but ARGs were observed to increase (Xi et al., 2009).

In this study, ARG occurrence patterns were evaluated in the POU water in three arid western U.S. recycled water distribution systems using quantitative polymerase chain reaction (qPCR). Depending on access, POE, POU biofilm, and soil irrigated with recycled water were also examined. Samples were also screened by qPCR for the potential presence of known waterborne pathogenic bacteria and indicators, *Legionella pneumophila*, *Escherichia coli*, and *Pseudomonas aeruginosa*. To simulate the effect of reused water for irrigation, a series of batch laboratory soil microcosm studies were performed to compare irrigation with secondary, chlorinated, and dechlorinated effluent from a representative conventional WWTP.

## MATERIALS AND METHODS

### WATER REUSE SYSTEMS

Samples were collected from three non-potable reclaimed wastewater distribution systems in the western U.S., together served by four WWTPs (**Table 1**). Water samples (-POE or -POU) were collected in sterile centrifuge tubes. Biofilm (-F) was collected with a sterile swab, and packed in a sterile centrifuge tube. A soil sample was collected from a field irrigated with reclaimed wastewater (-S), in a sterile centrifuge tube. All samples were shipped overnight on ice and stored frozen until extraction. Water samples were freeze-dried prior to DNA extraction (FreeZone Plus, Labconco, Kansas City, MO, USA).

**Table 1:** Summary of reclaimed WWTP tertiary treatment characteristics.

Aa^1^	Ab^1^	B	C
Capacity (Mgd)	6	4	1.1	0.6
Filtration	Media	Dual media	Carbon filter	Sand + activated carbon
carbon Disinfection	Chlorine	UV	Chlorine	Chlorine

*All WWTPs studied use conventional primary and secondary treatment processes*.

1 Aa and Ab are separate WWTPs that emit water to a commingled distribution system.

### BATCH MICROCOSMS

Aerobic, batch microcosms were prepared to investigate two water reuse scenarios on historically manured soil: (1) a single irrigation event (“batch irrigation”) and (2) repeated irrigation events (“periodic irrigation”). Soil was collected (upper 7.5–10 cm) in winter from historically manured corn fields near Virginia Tech campus using a soil probe. Soil was air dried and sieved (2 mm), and an aliquot was preserved for DNA extraction. For each study, microcosms were prepared in 250 mL flasks in triplicate with 50 g of soil and incubated at room temperature on a shaking table to maintain aerobic conditions. Slurry samples were collected weekly without sacrifice. Secondary, chlorinated, and dechlorinated WWTP effluents were collected before each irrigation treatment event from a representative 4.5 Mgd Domestic WWTP.

Batch irrigation soil was initially treated with 80 mL of freshly collected WWTP effluent fractions. Slurry samples (~0.4 g wet) were collected and an equal volume of deionized (DI) water was added to each flask to maintain soil moisture. WWTP effluent fractions (2–4 L) were filtered through 0.22 μm membrane and total DNA was extracted from the filter, as described below. The periodic irrigation soil was initially treated with 100 mL of freshly collected WWTP effluent fractions. Slurry samples (10 mL) were collected, centrifuged at 3,300 × *g* for 5 min, and 0.4 g of the pellet was used for DNA extraction. Fresh WWTP effluent fractions were added to the flasks to replace the volume removed during each sampling. WWTP effluent fractions (60 mL) were freeze-dried, as above, and DNA was extracted from the residuals.

### MOLECULAR TECHNIQUES

DNA was extracted from freeze-dried water/slurry, 0.4 g soil, or swabs using a FastDNA^®^ SPIN Kit for Soil (MP Biomedicals, Solon, OH, USA) and diluted 1:50 or 1:100 for the water reuse field study and 1:30 for the irrigation studies prior to downstream analysis. qPCR was performed to quantify 16S rRNA (Suzuki et al., 2000), *sul*1 (Aminov et al., 2001), *sul*2 (Aminov et al., 2001), *tet*(A) (Aminov et al., 2002), *tet*(O) (Aminov et al., 2001), *erm*F (Chen et al., 2007), *van*A (Dutka-Malen et al., 1995), *mec*A (McKinney and Pruden, 2012), *L. pneumophila*-specific *mip* (Nazarian et al., 2008), *E. coli*-specific *gad*AB (Chen et al., 2006), and *P. aeruginosa*-specific *ecf*X/*gyr*B (Anuj et al., 2009) genes for the water reuse field study. Reaction matrix and PCR protocols were as previously described (Ma et al., 2011; Wang et al., 2012). For the irrigation study, 16S rRNA, *sul*1, *sul*2, *tet*(O), and *tet*(W) genes were monitored. All standard curves of qPCR were constructed from serial dilutions of cloned genes ranging from 10^8^ to 10^2^ gene copies/μL. Samples were analyzed in triplicate with a standard curve and negative control included in each run. Limits of quantification with respect to sample volume varied depending on the volume processed and the dilution of DNA extract, ranging from -1.4 to 0.6 log_10_ gene copies/mL, 3.1–5.1 log_10_ gene copies per swab, and 0.5–2.5 log_10_ gene copies/g of soil. Additionally, cloning and sequencing of qPCR product was performed for assays that had not been validated previously (*van*A) to demonstrate specificity of PCR product (GenBank accession number KC792557–KC792573).

### STATISTICS

Cluster analysis was performed on transformed (square root) 16S rRNA gene normalized ARG profiles from the reclaimed wastewater systems and significance testing was carried out using the SimProf test in PrimerE (Plymouth, UK). To compare between wastewater treatment effluent fractions and treatments in the irrigation study, data was Box–Cox transformed. Transformed data were compared using ANOVA, and significant differences (*p* < 0.05) were determined using Tukey’s honest significance test, as implemented in R (http://www.r-project.org/). Multiple comparisons for the distribution system and irrigation studies were performed on Box–Cox transformed data using least square means comparison with a Satterthwaite estimation of degrees of freedom in SAS, again using Tukey adjustment for multiple comparisons. Linear modeling for the irrigation study data was performed in Microsoft Excel.

## RESULTS

### ARG OCCURRENCE PATTERNS IN RECYCLED WATER

**FIGURE 1:**
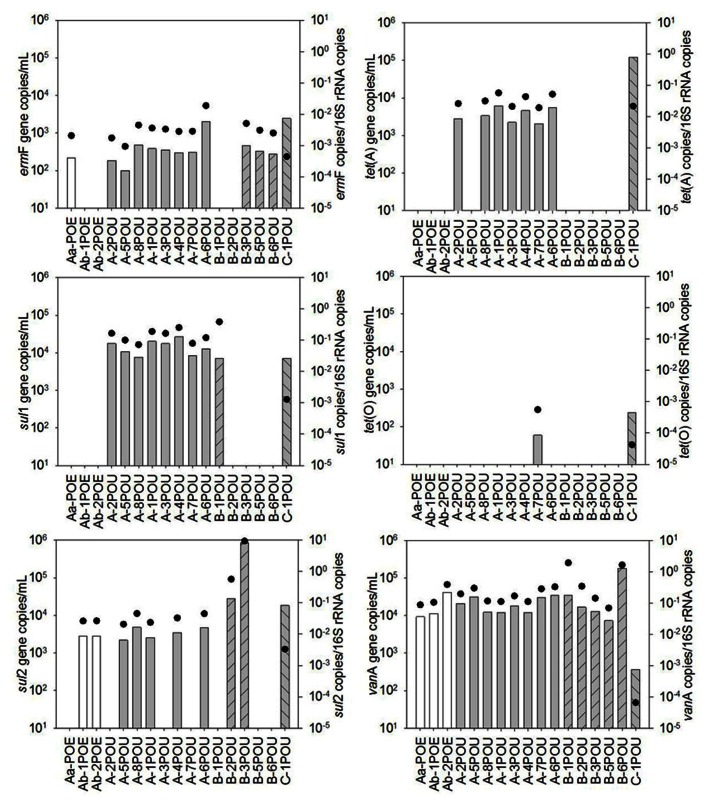
**Absolute (bars) and 16S rRNA gene-normalized (symbols) levels of ARGs in water samples collected at point of entry (POE) for WWTPs Aa and Ab and at point of use (POU) for systems A, B, and C, numbers differentiate between sample locations**.

**FIGURE 2:**
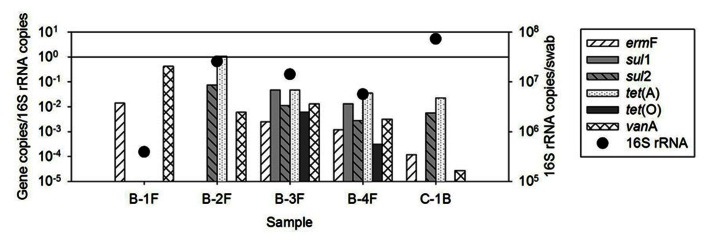
**Quantification of ARGs in biofilm samples available from recycled water distribution systems B and C.** Bars represent absolute ARG copies per swab, symbols represent 16S rRNA gene copies per swab.

Antibiotic resistance genes were detected in all water reuse samples (**Figures 1 and 2**). The most frequently detected ARGs were *van*A, *erm*F, and *sul*2 with frequencies of detection of 100, 73, and 65% (*n* = 23), respectively. *tet*(O) had the lowest frequency of detection (17%, *n* = 23) other than *mec*A, which was not detected in any of the samples. For System A, significant differences in ARG concentrations were observed between POE and POU for *sul*1 and *tet*(A), *p* < 0.001 for both. Concentrations of *van*A, *erm*F, and *sul*2, 16S rRNA, and *tet*(O) were not significantly different between POE and POU (*p* = 0.33–0.99).

In the irrigated soil sample (B-7S), 9.5, 7.3, 7.2, and 5.6 log_10_ gene copies/g of soil were quantified for 16S rRNA, *sul*1, *sul*2, and *van*A, respectively; *erm*F, *tet*(A), and *tet*(O) were below detection. Normalizing ARGs to 16S rRNA gene copy numbers indicated that *sul*1 and *sul*2 were one to two orders of magnitude lower and *van*A three to five orders of magnitude lower in the soil sample than observed in water and biofilm samples.

**FIGURE 3:**
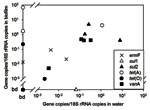
**Biofilm versus bulk water ARG copies normalized to 16S rRNA gene copies in available paired biofilm and water samples**. ARGs were below detection in several samples, indicated on the *x* and *y* axes as bd.

The occurrence of ARGs varied among the five biofilm samples examined, ranging from two to six classes of ARGs (**Figure 2**). However, individual ARG levels in the biofilm did not correlate with corresponding bulk water ARGs among the available paired samples (**Figure 3**). For example, the most abundant ARG, *tet*(A), in B-2F was below detection in B-2POU.

**FIGURE 4:**
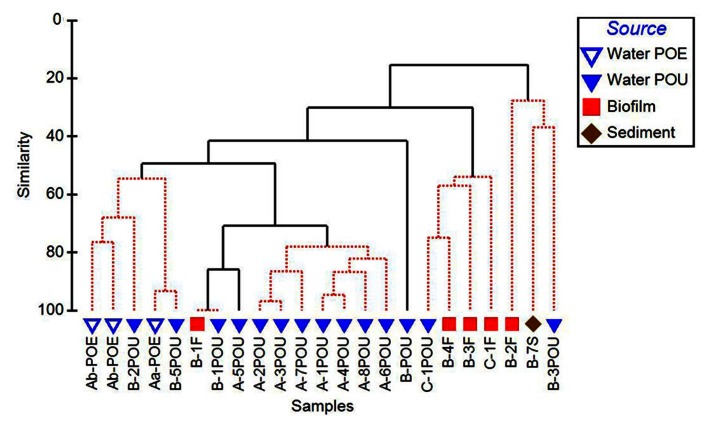
**Cluster analysis of 16S rRNA gene normalized ARG copy numbers, considerate of class and relative abundance of each ARG measured in the distribution systems (Aa, Ab, B, C) and across environmental matrices (POE, point of entry water; POU, point of use water; F, biofilm; S, soil)**. Solid branches indicate significantly different clusters (*p* < 0.05).

Cluster analysis performed on 16S rRNA gene normalized ARG profiles considered both the kinds of ARGs detected and their frequencies and resulted in several significantly different sample clusters (**Figure 4**). Generally, three distinct clusters were formed primarily by POE, WWTP-A POU, and WWTP-C POU samples, while the WWTP-B POU samples were interspersed among the three clusters, and a fourth cluster consisting only of WWTP-B samples. The three WWTP-A POE samples formed a significantly different cluster from POU samples in its corresponding distribution system (50% similarity). POU samples from System A (*n* = 7) formed a cluster with 78–96% similarity, except A-5POU which was significantly different (71%) and clustered with some samples from System B (*n* = 5). No patterns were observed in the biofilm and water clustering patterns for paired samples (*n* = 4). Biofilms did not cluster with one another nor did biofilm samples cluster with paired water sample with similarities ranging from 16% for F2 and F3 (significantly different) to 100% for F1 (no difference). The soil sample (B-7S) clustered with other B system biofilm and water samples, which were only 20% similar to the remainder of the samples selected.

### WATERBORNE PATHOGEN AND FECAL INDICATOR SCREENING

Quantitative PCR screening for *E. coli* and *L. pneumophila* through *gad*AB and *mip* resulted in positive detections for 48% of samples: 35 and 17% for *gad*AB and *mip*, respectively (**Table 2**). *ecrf*X/*gyr*B, corresponding to *P. aeruginosa*, were below detection in all samples.

**Table 2:** Log copies of *E. coli* (*gad*AB) and *L. pneumophila* (*mip*) specific genes per milliliter (POE and POU’s), swab (F’s), or gram soil (S).

Sample	*gad*AB	*mip*
Ab-POE	3.7	bd*
A-1POU	4.2	bd
A-2POU	5.1	1.8
A-3POU	4.4	bd
A-8POU	5.3	bd
B-1POU	bd	1.4
C-1POU	6.6	bd
B-1F	bd	4.6
B-2F	6.0	bd
C-1F	6.9	bd
B-7S	bd	5.6

* bd, below detection.

### ARGs DURING SIMULATED LAND APPLICATION

#### WWTP effluent fraction ARG loads

**FIGURE 5:**
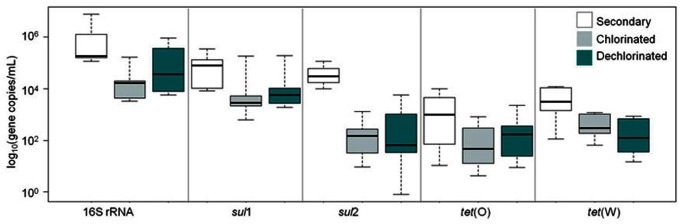
**Censored boxplot of 16S rRNA genes, *sul*1, *sul*2, *tet*(O), and *tet*(W) gene copies per milliliter in secondary (no fill), chlorinated (gray), and dechlorinated (blue) domestic WWTP effluents applied in the batch (2–4 L 0. 22 μm filtered) and periodic (60 mL freeze-dried) irrigation study (*n* = 6 *tet* genes, 7 *sul* and 16S rRNA genes)**.

Levels of ARGs in secondary, chlorinated, and dechlorinated WWTP effluent are compared in **Figure 5**. *sul*1 was the most frequently detected ARG (100%), followed by *sul*2 (71%), *tet*(W) (71%), and *tet*(O) (61%; *n* = 21). Comparing the ARGs and 16S rRNA gene levels across effluent fractions, secondary effluent was significantly higher than chlorinated and dechlorinated (both *p* < 0.0001) effluents, which were not significantly different from each other (*p* = 0.45). Comparing by gene across effluent fractions, secondary effluent and chlorinated effluent were significantly different in terms of 16S rRNA and *sul*2 gene copies (*p* < 0.0001 for both). Interestingly, when ARGs were normalized to 16S rRNA gene copies, significant differences were not observed among the secondary, chlorinated, or dechlorinated effluents (*p* = 0.29–0.91). This suggests no preferential destruction of specific gene types by chlorination.

#### Irrigation studies

**FIGURE 6:**
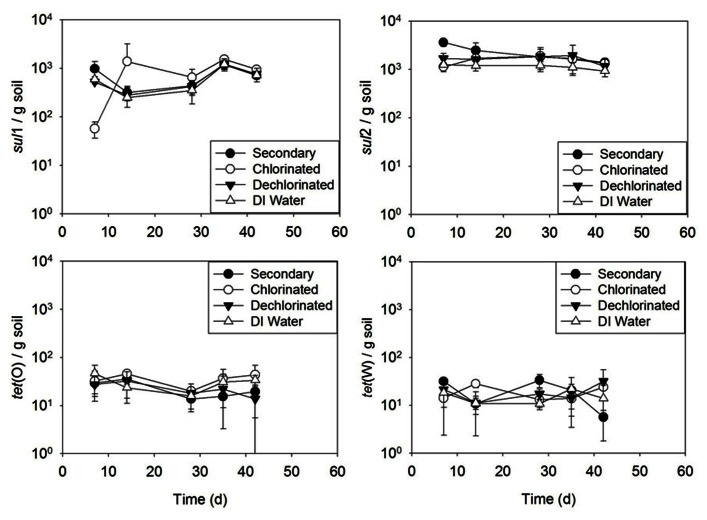
***sul*1, *sul*2, *tet*(O), and *tet*(W) genes in soils subject to one time (batch) irrigation with secondary, chlorinated, dechlorinated domestic WWTP effluents and deionized (DI) water, per gram of soil (slurry).** Error bars represent standard deviation of triplicate microcosms.

Results of the batch irrigation study (**Figure 6**) indicated no difference between soil irrigated with secondary, chlorinated, or dechlorinated effluent, or DI water in terms of ARG concentration with time for *sul*1, *sul*2, *tet*(O), or *tet*(W) (*p* > 0.405 for all, except for *sul*2 deionized *p* = 0.05 and secondary effluent *p* = 0.006).

**FIGURE 7:**
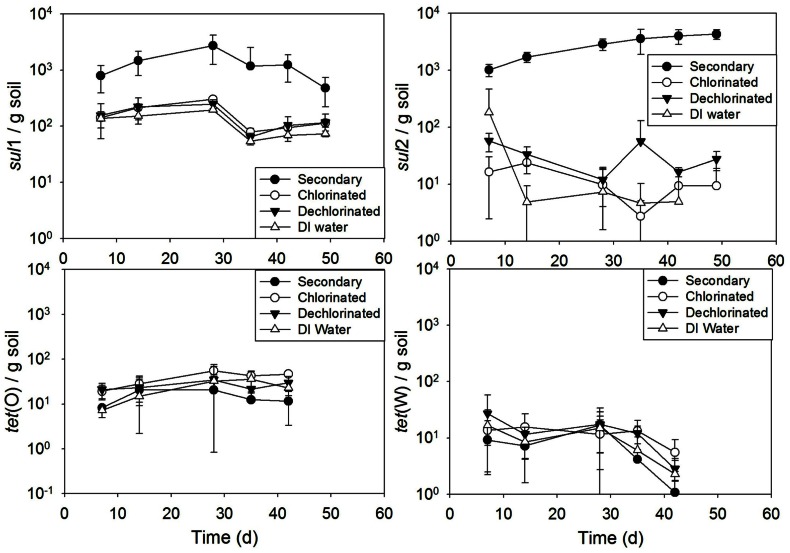
***sul*1, *sul*2, *tet*(O) and *tet*(W) genes in soils periodically irrigated with secondary, chlorinated, dechlorinated domestic WWTP effluents and deionized (DI) water, normalized to soil mass (wet weight)**. Error bars represent standard deviation of triplicate microcosms.

Soil periodically irrigated resulted in a significant increase in *sul*2 when receiving secondary effluent, compared to irrigation with the other water types (*p* ranging from 0.032 to *p* < 0.0001; **Figure 7**). Additionally, soil irrigated with secondary effluent had significantly higher *sul*1 copies than that irrigated with chlorinated effluent, dechlorinated effluent, or DI water (all *p* < 0.0001). No significant difference across time was observed among the irrigation water types for *tet*(O) (*p* = 0.13–1.0) or *tet*(W) (*p* = 0.74–1.0). Linear modeling of gene copies versus time revealed an increasing trend of *sul*2 copies with time in soil irrigated with secondary effluent (*R*^2^ = 0.92).

## DISCUSSION

This study explored the occurrence of several ARGs in three reclaimed water distribution systems in the U.S. Given limited access to such systems, only one sampling event was possible. Nonetheless, the results provide important baseline information to support future research, including insight into the kinds of ARGs, bacteria, and applications that may be of concern. To the knowledge of the authors, this is the first study specifically investigating the potential for ARGs to persist or amplify within the reclaimed water distribution pipes.

### ARG OCCURRENCE IN RECYCLED WATER DISTRIBUTION SYSTEMS

Several ARGs were detected at the POU in this study, many of which were below detection at the POE. This highlights the need to consider microbiological processes occurring in reclaimed water distribution systems that may be contributing to ARG amplification and suggests that focus on the water quality at the POU may be the most appropriate for assessing risk. Re-growth is a well-known phenomenon even in drinking water distribution systems (Xi et al., 2009), and has also recently been documented in a recycled water system (Ryu et al., 2005).

Several studies have documented levels of ARGs in WWTP effluents, providing a reference for comparison. Generally, *erm*F, *sul*1, *sul*2, *tet*(A), and *tet*(O) levels measured in the POE, POU, and WWTP effluent applied in the irrigation study were below or at the lower end of ranges reported by others in WWTP effluents (Kim et al., 2010; Czekalski et al., 2012; Negreanu et al., 2012). However, the irrigated soil in this study carried higher levels of *sul*1 (7.3 log_10_ copies/g) and *sul*2 (7.2 log_10_ copies/g) than reported by others in a field study of irrigation with recycled water (5.1–6.7 and 3.5–4.7 log_10_ copies/g, respectively; Negreanu et al., 2012).

The occurrence of *van*A is of particular interest given that it confers resistance to vancomycin, a last-resort life-saving antibiotic. Vancomycin is commonly prescribed to treat methicillin-resistant *Staphylococcus aureus* (MRSA) infections, but has been losing effectiveness due to increased resistance among staphylococci (Stevens, 2006). *van*A was detected in every sample in this study using primers targeting a 732-bp product. Interestingly, using *van*A primers designed for longer target products (1030 bp; Clark et al., 1993), *van*A was detectable at several POU sites, but not in any POE samples (data not shown). Detection differences between the two PCR primer sets may indicate that *van*A was partially damaged during disinfection, preventing amplification of longer PCR products. Long-amplicon PCR has recently been demonstrated to provide enhanced detection of DNA damage events (McKinney and Pruden, 2012). Few studies have reported detection of *van*A in environmental samples, providing little reference for comparison. However, *van*A has been reported in drinking water biofilms and wastewater (Schwartz et al., 2003), but was below detection in wastewater reclaimed for groundwater recharge environments (Böckelmann et al., 2009). To the knowledge of the authors, this is the first report of the presence of *van*A in distributed recycled water.

### MOLECULAR DETECTION OF WATERBORNE PATHOGENS AND INDICATORS

Opportunistic pathogens residing in water systems, such as *L. pneumophila* and *P. aeruginosa*, are now the primary source of waterborne disease outbreak in developed countries (Brunkard et al., 2011). However, there is a need for epidemiological studies to better quantify the precise contributions of various water systems to human disease (Pruden et al., 2013). Of interest to the present study was whether such organisms may be present in recycled water, which could be of special concern for bacterial pathogens because they are capable of developing antibiotic resistance. *P. aeruginosa* is an example of an opportunistic pathogen that colonizes taps and is prone to multi-antibiotic resistant forms (Trautmann et al., 2005). *E. coli* was also of interest as a fecal indicator.

The *mip* gene, specific to *L. pneumophila*, was detectable at levels comparable to those recently observed in chloraminated drinking water distribution systems (Wang et al., 2012). Thus, there could be concern for aerosolization of *L. pneumophila* during spray irrigation. Future research is suggested to more closely examine this potential transmission pathway. *gad*AB, specific to *E. coli*, detected at POU in this study, was comparable to levels previously observed in manure runoff (4.6–4.9 log_10_ copies/mL, assuming one gene copy per cell) and higher than previously observed in WWTP effluent (2.74 log_10_ copies/mL) (Grant et al., 2001). This combined with the observation of *gad*AB below detection at POE, but detectable at POU, is further evidence of bacterial re-growth within reclaimed water distribution systems.

### EFFECT OF DISINFECTION ON ARGs

Chlorination is commonly applied to WWTP effluents during warm seasons, in which case the effluent must be dechlorinated prior to discharge. Chlorination had a significant impact on 16S rRNA and *sul*2 gene copies, which is consistent with the expectation that chlorination would have a moderate reactivity with nucleic acids (Dodd, 2012). Although occasionally ARGs detected in dechlorinated effluent were not detected in chlorinated effluent, the levels of detected genes were not significantly different. The levels of ARGs in secondary, chlorinated, and dechlorinated WWTP effluent fractions were comparable to those detected at the POU in the field study (**Figure 5**).

### ARG FATE DURING WWTP EFFLUENT LAND APPLICATION

In the lab study, periodic irrigation with secondary effluent increased the prevalence of *sul*1 and *sul*2 in historically manured soil compared to soil irrigated with chlorinated, dechlorinated, or DI water. This could be due to direct inputs of extracellular ARGs, intracellular ARGs, or horizontal gene transfer to native soil bacteria (Dodd, 2012). Interestingly, *tet*(O) or *tet*(W) levels in the soil slurry were not affected by irrigation. This highlights that different ARGs have different environmental fates, as has been observed recently with respect to *sul*1 and *tet*(W) in a watershed-scale study (Storteboom et al., 2010; Pruden et al., 2012). Differences in ARG fate likely relate to host properties and their overall propensity for horizontal gene transfer. Because 16S rRNA gene copy levels were relatively consistent with time across the soil irrigation treatments (data not shown), changes in total bacterial population sizes were not likely a factor in the observed differences.

A recent field study carried out in Israel suggested that irrigation does not significantly affect the soil microbiome, and increases were not observed in *sul*1, *sul*2, *tet*(O), *erm*F, or *erm*B in soil subjected to long-term (6–12 years) irrigation with secondary effluent compared to freshwater irrigation (Negreanu et al., 2012). Given the difference in controls, irrigation frequency, soil type, climate, and wastewater chemistry, direct comparison between the studies is difficult. It is suspected that the difference in soil moisture and incubation time may likely be an important difference. Negreanu et al. (2012) irrigated with 4 L of water/m^2^ soil/day which experienced a combination of infiltration, run off, and evaporation. In this study, soil-water slurries were used with irrigation waters that incubated with soil rather than infiltrating, allowing for greater contact time. Therefore, the microcosm results presented here may indicate that irrigating at high rates with secondary effluent may still result in amplified soil ARGs. The present study is consistent with the observation of increased ARG copies directly under irrigation drippers compared to soils 50 m from drippers (Negreanu et al., 2012). Further, soil type has been noted to be a critical factor in determining the level of impact of land application of biosolids containing ARGs (Munir and Xagoraraki, 2011), and may also affect the fate of ARGs applied by irrigation.

Understanding land use scenarios that affect soil ARGs is of utmost importance given that the resistome of multi-drug resistant soil bacteria was recently shown to match that from diverse human pathogens (Forsberg et al., 2012). Heavy irrigation with secondary effluent is shown here to be capable of increasing soil ARGs. In practice, given that ARG prevalence can increase between POE and POU within distribution systems, increased ARG levels in irrigation waters and therefore soils are expected. Given that spray irrigation of recreational fields with treated wastewater is common practice, there is considerable potential for human contact with aerosols and soil.

## CONCLUSION

This study brings to light the occurrence of ARGs at the POU in recycled water irrigation systems, including *van*A, which is of significant concern to human health. Differences between POE and POU ARG occurrences underscore the need to take into consideration re-growth that occurs in the distribution system when estimating overall exposure and risk. Based on the lab microcosm study, amplified levels of ARGs in soil irrigated with recycled water is possible. Molecular data in this study also indicates the potential presence of waterborne bacterial pathogens, such as *L. pneumophila*. As ARGs are emerging contaminants, risk assessment is in its infancy and no guidance yet exists on safe levels (Pruden, 2011). In addition to direct contact with water and aerosols during recreational activity, a greater concern may be overall contribution to the global pool of antibiotic resistance and ultimately reducing the effectiveness of available antibiotics for treating human disease.

## Conflict of Interest Statement

The authors declare that the research was conducted in the absence of any commercial or financial relationships that could be construed as a potential conflict of interest.
